# Comparative Study on Inhibition of Pancreatic Cancer Cells by Resveratrol Gold Nanoparticles and a Resveratrol Nanoemulsion Prepared from Grape Skin

**DOI:** 10.3390/pharmaceutics13111871

**Published:** 2021-11-05

**Authors:** Baskaran Stephen Inbaraj, Leng-Huei Hua, Bing-Huei Chen

**Affiliations:** 1 Department of Food Science, Fu Jen Catholic University, New Taipei City 24205, Taiwan; 138547@mail.fju.edu.tw (B.S.I.); m3426531@gmail.com (L.-H.H.); 2 Department of Nutrition, China Medical University, Taichung 40401, Taiwan

**Keywords:** grape skin, resveratrol-gold nanoparticle, resveratrol nanoemulsion, pancreatic cancer cells BxPC-3, western blotting

## Abstract

Resveratrol, a phenolic compound possessing vital biological activities such as anti-cancer, is present abundantly in grape skin, a waste produced during the processing of grape juice. The objectives of this study were to prepare resveratrol-gold nanoparticles and a resveratrol nanoemulsion from grape skin and study their inhibition effects on pancreatic cancer cells BxPC-3. The spherical-shaped citrate gold nanoparticles (GNPs) and resveratrol-gold nanoparticles (R-GNPs) were, respectively, prepared with a surface plasmon resonance peak at 528 and 538 nm, mean particle size of 20.8 and 11.9 nm, and zeta-potential at −32.7 and −66.7 mV, by controlling an appropriate concentration of citrate/resveratrol and gold chloride as well as stirring time and temperature. The resveratrol nanoemulsion, composed of soybean oil, Tween 80, and sucrose fatty acid ester in glycerol and water, possessed a high storage stability with a mean particle size of 14.1 nm, zeta-potential of −49.7 mV, and encapsulation efficiency of 95.5%. An antiproliferation study revealed that both R-GNPs and resveratrol nanoemulsion could effectively inhibit the growth of pancreatic cancer cells BxPC-3, with the latter showing a higher inhibition effect. Western blot analysis implied that both can down-regulate expressions of cyclin A, cyclin B, CDK1, and CDK2 and up-regulate expressions of p53 and p21, accompanied by enhancing cytochrome C expression, decreasing BcL-2 expression, increasing Bax expression, and leading to the elevation of caspase-8, caspase-9, and caspase-3 activities for cell apoptosis execution. Future research is needed to study the inhibition of pancreatic tumors in vivo by R-GNPs and resveratrol nanoemulsions.

## 1. Introduction

Grape, one of the major fruits produced in the world, is rich in many vital nutrients such as vitamins and minerals as well as phytochemicals such as phenolic acids and flavonoids, both of which have been demonstrated to possess important biological activities such as antioxidation, anti-inflammation, cardioprotection, anticancer, and anti-bacteria [1]. Of the various phenolic acids and flavonoids, resveratrol was also shown to exhibit many physiological functions including anti-inflammation, anticancer, neuroprotection, cardioprotection, anti-diabetes, anti-dyslipidemia, anti-aging and anti-rheumatism [2,3]. However, resveratrol can undergo a great loss during the processing of grapes into grape juice, one of the most imperative commercial beverage products on the market worldwide. Nevertheless, grape skin, a by-product obtained during the processing of grape juice and often discarded after processing, was shown to contain a significant amount of resveratrol at 20–100 µg/g based on dry weight [4]. Thus, it will be of great advantage to the food industry if resveratrol in grape skin can be extracted and developed into functional foods or even a botanic drug.

Resveratrol, naturally present in trans and glycosyl form, can be converted to cis-resveratrol after exposure to light, which can be more susceptible to heat and light degradations [5]. Moreover, the bioavailability of cis-resveratrol in rats was reported to be lower than that of trans-resveratrol [6]. Also, because of low aqueous solubility, the bioavailability of both trans- and cis-resveratrol in vivo is quite low, and thus the biological activity can be affected greatly [5]. To remedy this problem, many techniques such as nanoemulsions, liposomes, and polymeric nanoparticles have been developed to encapsulate resveratrol to enhance its aqueous solubility and stability in vivo so that the biological activity of resveratrol can be improved substantially [7].

Accordingly, emulsions can be classified into nanoemulsions, microemulsions, and macroemulsions, with the mean particle size ranging from 10 to 100 nm, 2 to 100 nm, and 1 to 100 µm, respectively [8]. Among the various emulsions, both oil-in-water (O/W) and water-in-oil (W/O) types are frequently prepared for their wide applications in the field of foods, cosmetics, drugs, and environments. Compared to microemulsions, nanoemulsions have been reported to be more kinetically stable [8], and both can be used as drug delivery systems for the encapsulation of unstable bioactive compounds to enhance biological activity [9]. Furthermore, both microemulsions and nanoemulsions are simple ternary systems composed of water phase, oil phase, and surfactants, with co-solvent and co-surfactant sometimes being used to increase the encapsulation efficiency and stability of both systems [9].

Of the various nanomaterials used in biomedical applications, gold is the most popular one due to its capability in conjugation with drugs to act as a carrier for drug delivery and a contrast agent for imaging enhancement [10]. More importantly, the conjugation of gold nanoparticle with a specific cancer cell surface receptor such as folate has been shown to enhance the efficiency of cancer treatment through active targeting [11]. In addition to active targeting, it is also possible to strengthen cancer therapy efficiency through the conjugation of gold nanoparticles with lipid-based microemulsion or nanoemulsion containing bioactive compounds. For instance, in a previous study, Huang et al. [9] prepared a nanogold-lycopene nanoemulsion with a mean particle size of 21.3 nm by transmission electron microscope (TEM) analysis, and they demonstrated a substantially higher synergistic effect of this nanoemulsion in inhibiting the growth of colon cancer cells HT-29 than nanogold or lycopene when used alone. More specifically, the TEM image revealed that numerous nanoemulsion-filled vacuoles invaded cytosol and converged into mitochondria, resulting in an abnormally elongated morphology with reduced cristae and matrix contents, demonstrating a possible passive targeting effect in treating colon cancer through the enhanced permeability and retention (EPR) effect [9].

In view of the great impact of nanocomposites on possible treatments of cancer, this study was undertaken to isolate resveratrol from grape skin for preparation of a resveratrol nanoemulsion to explore its inhibition effect on the growth of pancreatic cancer cells BxPC-3. In addition, gold-resveratrol nanoparticles were also prepared to compare their inhibition effect on pancreatic cancer cells BxPC-3.

## 2. Materials and Methods

### 2.1. Materials 

Grape skin (10 kg) was collected from a local fruit juice shop located at Hsinchuang district, New Taipei City, Taiwan. Prior to experiments, the grape skin was freeze-dried and ground into powder (2 kg). Trans-resveratrol standard was procured from Sigma-Aldrich Co. (St. Louis, MO, USA). The HPLC-grade solvents including acetonitrile, acetone, and methanol were from Merck Co. (Darmstadt, Germany). Ethyl acetate, hydrochloric acid, sodium bicarbonate, and 99% ethanol were also from Sigma-Aldrich Co. Deionized water was made using a water purification system from Millipore Co. (Bedford, MA, USA). Sucrose fatty acid ester (S-1570) was from Chen-Fang Co. (Taipei, Taiwan). Both Tween 80 and glycerol were from Yu-Pa Co. (Taipei, Taiwan). Gold chloride trihydrate solution, potassium dihydrogen phosphate, trisodium citrate, sodium carbonate, and Folin–Ciocalteu phenol reagent were also from Sigma-Aldrich Co. A Gemini C18 110A column (250 *×* 4.6 mm ID, particle size 5 µm) was from Phenomenex Co. (Torrance, CA, USA).

### 2.2. Cell Culture

Both pancreatic endothelial cells (MS1) and pancreatic cancer cells (BxPC-3) were from the Bioresource Collection and Research Center, Taiwan Food Industry Research and Development Institute (Hsinchu, Taiwan). Dulbecco’s modified Eagle medium (DMEM), Rosewell Park Memorial Institute (RPMI) medium 1640, fetal bovine serum (FBS), 4-(2-hydroxyethyl)-1-piperazineethanesulfonic acid (HEPES) solution, phosphate buffered solution (PBS), sodium pyruvate solution, Hank’s balanced salt solution (HBSS), Dulbecco’s phosphate buffer solution (DPBS), and 0.25% trypsin-EDTA were all from Hyclone Co. (Logan, UT, USA). Bovine serum albumin (BSA), sodium dodecyl sulfate (SDS), *β*-mercaptoethanol, bromophenol, 0.4% trypan blue, thiazolyl blue tetrazolium bromide (MTT), and ethylenediaminetetraacetic acid (EDTA) were from Sigma-Aldrich Co. Dimethyl sulfoxide (DMSO) was from Merck Co. (Taipei, Taiwan). Ammonium persulfate (APS), glycine, *N,N,N′,N′*-tetramethylethylenediamine (TEMED), tris(hydroxymethyl)-aminomethane (Tris), and 30% acrylamide were from USB Co. (Cleveland, OH, USA). Both Bradford reagent (protein assay) and Clarity Western ECL substrate were from Bio-Rad Co. (Hercules, CA, USA). HyBlock blocking buffer was from Han-Hsin Co. (Taipei, Taiwan). Caspase-3, caspase-8, and caspase-9 assay kits were from Bio Vision Co. (Milpitas, CA, USA). Primary antibodies including cytochrome C, p21, BcL-2, p53, cyclin B, cyclin A, CDK1, and *β*-actin were from Biotools Co. (New Taipei City, Taiwan), while Bax and CDK2 were from Santa Cruz Biotechnologies Co. (Santa Cruz, CA, USA). Secondary antibodies including anti-mouse-IgG-HRP and anti-rabbit-IgG-HRP were also from Biotools Co. (New Taipei City, Taiwan).

### 2.3. Instruments

The HPLC-MS instrument, composed of a degasser (G1379B), quaternary pump (G1312B), autoinjector (G1329B), column temperature controller (G1316B), photodiode array detector (PDA) (G1315C), and single quadrupole mass spectrometers (6130) with atmospheric pressure chemical ionization (APCI) detection mode, was from Agilent Technologies (Santa Clara, CA, USA). The high-speed centrifuge (Sorvall RC5C) was from Du Pont Co. (Wilmington, DL, USA), while the microcentrifuge (Fresco 21) was from Thermo Fisher Scientific Co. (San Jose, CA, USA). The rotary evaporator (N-1200A) was from Eyela Co. (Tokyo, Japan). The sonicator (DC400H) was from Asiatek Co. (Taipei, Taiwan). The dynamic light scattering instrument was from Brookhaven Co. (Holtsville, NY, USA). The zeta potential analyzer was from Horiba Scientific Co. (Kyoto, Japan). The transmission electron microscope (TEM) was from JEOL Co. (Tokyo, Japan). The inverted microscope (Eclipse TS 100) was from Nikon Co. (Tokyo, Japan). The ELISA reader was from Molecular Devices Co. (Sunnyvale, CA, USA). The multimode microplate reader (Infinite 200 PRO) was from TECAN Co. (Zürich, Switzerland). The electrophoresis system was from Bio-Rad Laboratories (Hercules, CA, USA). The spectrophotometer (DU70) was from Beckman Co. (Fullerton, CA, USA). The luminescence/fluorescence imaging system (MGIS-21-C2-1M) was from Top Bio Co. (New Taipei City, Taiwan). The CO_2_ incubator (MCO-20AIC) was from Panasonic Co. (Osaka, Japan).

### 2.4. Extraction of Resveratrol from Grape Skin

A method based on Wang et al. [12] was modified to extract resveratrol from grape skin. Initially a 40 g sample of grape skin powder was mixed with 400 mL of 95% ethanol, after which this mixture was extracted in a 75 °C water bath for 1 h and this procedure was repeated twice. After centrifugation at 3800× *g* for 30 min, the supernatant was collected and evaporated to dryness, followed by dissolving in 150 mL of deionized water, sonicating for 20 min, filtering through a filter paper, adjusting pH to 1 with 1 M hydrochloric acid, and stirring for 3 h. Then, the extractant (50 mL) was collected and mixed with ethyl acetate (50 mL) in a separatory funnel for separation into two layers and the organic layer was collected. This procedure was repeated 3 times and all the organic layers were pooled in a separatory funnel, followed by adding 5% sodium bicarbonate solution (pH 8–9) at a volume ratio of 1:1, washing until the organic layer became colorless, evaporating to dryness, dissolving in 0.5 mL of methanol, and filtering through a 0.22 µm membrane filter for HPLC analysis. Trans-resveratrol and cis-resveratrol in the grape skin were separated, identified, and quantified using a method described in a previous study [13].

### 2.5. Preparation of Resveratrol Nanoemulsion

Initially 5 mL of resveratrol extract was collected from the organic layers shown above and evaporated to dryness under nitrogen, followed by adding 0.1 g soybean oil (1%) containing vitamin E (1%), stirring for complete mixing, adding 0.7 g Tween 80 (7%) and 0.4 g sucrose fatty acid ester in glycerol (4%) and stirring again. Then 8.8 g deionized water (88%) was added and the mixture was sonicated for 1 h to obtain a transparent resveratrol nanoemulsion containing resveratrol at 200 mg/mL.

### 2.6. Characteristics Determination of Resveratrol Nanoemulsion

For particle size and polydispersity determination, a portion (10 µL) of the resveratrol nanoemulsion was collected and diluted 100-fold with a potassium dihydrogen phosphate solution, after which a portion was poured into a colorimetric tube for determination by a dynamic light scattering instrument (DLS) with the BIC particle sizing 90Plus software system. For zeta potential determination, a portion (10 µL) of the resveratrol nanoemulsion was diluted with deionized water 100-fold, and a 300 µL sample was placed in an analysis cell for determination by a zeta potential analyzer. The TEM image of the resveratrol nanoemulsion was determined by diluting the resveratrol nanoemulsion with deionized water 200-fold and a portion (20 µL) was placed on a copper grid for sinking for 1 min, followed by removing excess sample with a filter paper, negative staining with 2% phosphotungstic acid for 45 s, removing excess staining agent, drying in a desiccator, and observing the image under 120 kV by enlarging 3 × 10^5^ times. Then, both the size and shape of the resveratrol nanoemulsion were obtained. Also, the encapsulation efficiency was determined by collecting a portion (100 µL) of the resveratrol nanoemulsion and diluting it with deionized water 10 times, followed by pouring into a centrifuge tube containing a 3 kDa dialysis membrane, centrifuging at 12,000× *g* for 20 min (25 °C), collecting the lower layer (200 µL) containing free resveratrol, evaporating to dryness, and dissolving in methanol for HPLC analysis of resveratrol [13]. Then, the encapsulation efficiency of resveratrol was obtained by using a formula described in a previous study [14].

### 2.7. Stability of Resveratrol Nanoemulsion

The resveratrol nanoemulsion was stored at 4 °C for 90 days, during when a portion was collected every 15 days for determination of particle size and polydispersity index by DLS, as well as zeta potential by a zeta potential analyzer. Similarly, a portion (200 µL) was collected and placed in a 40 °C, 60 °C, 80 °C, and 100 °C water bath separately for heating for 0.5, 1, 1.5, or 2 h, after which both the particle size and polydispersity index of each sample were determined by DLS, while zeta potential was analyzed by a zeta potential analyzer.

### 2.8. Preparation of Gold Nanoparticle (GNPs) and Resveratrol-Gold Nanoparticle (R-GNPs)

A method based on Kimling et al. [15] and Inbaraj et al. [16] was modified to prepare the GNPs. One mL of gold (III) chloride trihydrate solution was mixed with 1 mL of deionized water in a tube, followed by heating on a plate at 75 °C and stirring for 20 min at 1000 rpm Then, 3 mL of 5 mM sodium citrate solution was added with continued heating and stirring until the solution color turned red, and 5 mL of the GNPs was obtained. Likewise, a method based on Thipe et al. [17] was modified to prepare the R-GNPs. Two mL of gold(III) chloride trihydrate solution was mixed with 2 mL of deionized water in a tube, followed by heating on a plate at 40 °C and stirring at 1000 rpm for 20 min. Then, 1 mL of resveratrol standard solution (5 mM) was added with continued heating and stirring until the solution color turned red, and 5 mL of R-GNPs was prepared.

### 2.9. Characteristics Determination of GNPs and R-GNPs

For determination of the absorption spectra, gold(III) chloride trihydrate solution, GNPs and R-GNPs were each diluted with deionized water 10-fold, after which 2 mL of each sample was collected in a colorimetric tube for determination by a spectrophotometer. For zeta potential determination, 100 µL of the GNPs and R-GNPs were collected separately and diluted with deionized water 10-fold, after which 300 μL of each sample was poured into a cell for determination by a zeta potential analyzer. Also, for TEM imaging, samples of the GNPs and R-GNPs were collected separately and diluted with deionized water 50 times, after which 20 μL of each sample was placed on a copper grid for sinking for 1 min, followed by removing excess sample with a filter paper, drying in a desiccator, and observing the image under 120 kV by enlarging 3 *×* 10^5^ times. The resveratrol concentration in the R-GNPs was determined based on a method by Thipe et al. [17] with some modifications. Initially, six concentrations of resveratrol standard (7.8, 15.6, 31.2, 62.5, 125, and 250 ppm) were prepared, after which 0.5 mL of each and R-GNPs were collected separately in a 15 mL centrifuge tube, followed by adding 0.5 mL of Folin–Ciocalteu phenol agent, mixing thoroughly, reacting in the dark for 5 min, adding 1 mL of sodium carbonate solution (7%), mixing thoroughly, reacting in the dark for 1 h, and measuring absorbance at 750 nm with an ELISA reader. Then, the resveratrol concentration in the R-GNPs was obtained based on the standard curve of resveratrol standard.

### 2.10. Cell Culture Experiment

Pancreatic cancer cells BxPC-3 were cultured in RPMI 1640 medium containing 2 mM L-glutamine, 1.5 g/L sodium bicarbonate, 1.0 mM sodium pyruvate, 4.5 g/L glucose, 10 mM HEPES, and 10% FBS, while pancreatic endothelial cells MS1 was cultured in DMEM medium containing 4 mM l-glutamine, 1.5 g/L sodium bicarbonate, 4.5 g/L glucose, 1.0 mM sodium pyruvate and 5% FBS. After collection from liquid nitrogen, cells were thawed at 37 °C and cells containing 7% DMSO were added to a 10 cm plate, followed by adding 10 mL of medium and culturing in an incubator with a temperature of 37 °C, relative humidity of 100%, and carbon dioxide at 5%. After cells reached a confluency of 80–90%, PBS was added for washing 2–3 times and then 2 mL of 0.25% trypsin-EDTA added for reaction in an incubator for 3–5 min. Next, 2 mL of medium was added and centrifuged at 1500× *g* for 4 min, followed by removing the supernatant, adding 1 mL of medium, and collecting a portion of the cells cultured in a new medium for further counting under a microscope.

#### MTT assay

Cells containing BxPC-3 or MS1 were seeded in a 96-well plate with each well containing 3 *×* 10^3^ cells and cultured 48 h for cell adhesion, after which the medium was removed and replaced with different concentrations (1.25, 2.5, 5, 10, 15, and 20 µg/mL) of the GNPs, R-GNPs, resveratrol standard, or resveratrol nanoemulsion. After 48 h of incubation, the medium was removed and MTT (200 µL) added for reaction for 1 h, followed by adding 100 µL of DMSO, stirring to dissolve purple crystal, and measuring absorbance at 570 nm with an ELISA reader for the determination of cell viability [9].

### 2.11. Western Blotting

BxPC-3 cells (1 *×* 10^6^) were seeded in a 10 cm plate and cultured overnight for cell adhesion. Then, the medium was removed and replaced with three concentrations (10, 15, and 20 µg/mL) of the resveratrol nanoemulsion or R-GNPs for incubation for 48 h. Following medium removal and washing with PBS, 2 mL of 0.25% trypsin-EDTA was added for centrifugation at 1500× *g* for 4 min. Then, the supernatant was removed, followed by adding 100 µL of cell lysis buffer, standing overnight at −20 °C, centrifuging again at 12,000 rpm for 30 min (4 °C), and collecting the supernatant as the protein extract. For protein quantitation, eight concentrations of bovine serum albumin (BSA) standards (0, 100, 200, 400, 500, 600, 800, and 1000 µg/mL) were prepared and 10 µL of each was collected in a 96-well plate, followed by adding 200 µL of Bradford reagent for reaction for 5 min, measuring absorbance at 595 nm with an ELISA reader, preparing the standard curve by plotting concentration against absorbance, and finally the protein in the cell extract was quantified based on the standard curve. Next, a sample of protein extract was collected and sample buffer added for reaction in a 98 °C water bath for 10 min. After cooling on ice, the samples were added in a tank for protein separation with the upper voltage being 90 V (20 min) and the lower voltage being 110 V (80 min). Then, the PVDF membrane was soaked in methanol for 10 min, followed by soaking in transfer buffer, incubating at 4 °C for 3 h (100 V), soaking in blocking buffer, removing blocking buffer, and washing with TBST 5 times for 15 min each. The primary antibodies was added as follows: cytochrome C (1:1250), p21 (1:500), BcL-2 (1:1250), p53 (1:500), cyclin B (1:500), cyclin A (1:500), CDK1 (1:500), Bax (1:500), CDK2 (1:500), and *β*-actin (1:1250). Following reaction at 4 °C overnight, TBST was added for washing 5 times for 15 min each and the horseradish peroxidase (HRP)-conjugated secondary antibody (IgG) was added for reaction at 4 °C for 2 h, followed by washing with TBST 5 times for 15 min each, adding ECL reagent for reaction, and detection by a fluorescence imaging system. Following Western blotting, a band was circled and the value determined automatically by a fluorescence imaging software system (ImageJ), followed by dividing by a marker value to obtain a number. Then, the number was divided by the control value to obtain the expression values.

### 2.12. Activities of Caspase-3, Caspase-8, and Caspase-9

All the activities of caspase-3, caspase-8, and caspase-9 were determined by a fluorometric assay kit. In brief, the caspase-3 activity was determined by collecting cell extract (25 µL), adding 50 µL of 2X reaction buffer containing 5 µL of Ac-DEVD-AMC substrate, reacting in a 37 °C water bath for 1 h, and transferring to a 96-well plate for measuring absorbance with an excitation wavelength at 400 nm and emission wavelength at 505 nm by a multimode microplate reader. Similarly, the activities of caspase-8 and caspase-9 were determined by collecting cell extract (25 µL), followed by adding 50 µL of 2X reaction buffer containing 10 mM DTT and 5 µL of 1 mM IETD-AFC substrate for caspase-8 or 1 mM LEHD-AFC substrate for caspase-9, reacting in a 37 °C water bath for 1 h, transferring to a 96-well plate, and measuring absorbance with an excitation wavelength at 400 nm and emission wavelength at 505 nm by a multimode microplate reader. 

### 2.13. Statistical Analysis

All the data were subjected to statistical analysis by SAS [18] for ANOVA analysis and Duncan’s multiple range test for significance in mean comparison (*p* < 0.05).

## 3. Results and Discussion

### 3.1. Analysis of Trans-Resveratrol and Cis-Resveratrol in Grape Skin

Based on the extraction and HPLC method described in a previous study [13], a total of five trans-resveratrol and related stilbenes in grape skin were identified and quantified, with cis-piceid present in the largest amount (4.185 µg/g), followed by trans-piceid (1.643 µg/g), trans-resveratrol (1.539 µg/g), ε-viniferin (1.056 µg/g), and cis-resveratrol (0.293 µg/g). 

### 3.2. Characteristics of Resveratrol Nanoemulsion

**Figure 1:**
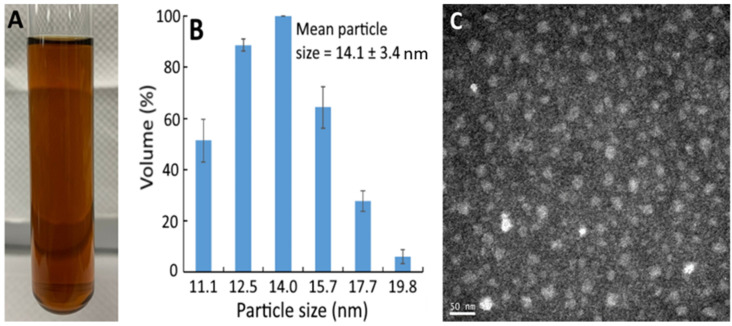
Appearance of resveratrol nanoemulsion prepared from grape skin (**A**) and its particle size distribution as determined by dynamic light scattering method (**B**) as well as the transmission microscopic image obtained at 120 kV (**C**). The resveratrol nanoemulsion with a mean particle size of 14.1 ± 3.4 nm and polydispersity index of 0.296 was prepared by mixing 1% soybean oil, 7% Tween 80, 4% sucrose fatty acid ester in glycerol, and 88% water.

Figure 1A shows the bright red appearance of the resveratrol nanoemulsion, with the mean particle size and polydispersity index (PDI) being 14.1 nm (Figure 1B) and 0.296, respectively, as determined by DLS. This outcome implies a monodispersion of nanoparticles in this resveratrol nanoemulsion because of low PDI. Also, the TEM image shows round-shaped nanoparticles with the mean particle size of 15 nm (Figure 1C), which was slightly higher than that determined by DLS. In several previous studies, Sessa et al. [19] prepared a resveratrol nanoemulsion composed of peanut oil, ethanol, lecithin, sucrose fatty acid ester, and deionized water with the mean particle size of 137.5 nm and PDI of 0.22, respectively. Similarly, Kumar et al. [20] prepared a resveratrol nanoemulsion composed of ethyl oleate, lecithin, Tween 80, and deionized water with the mean particle size and PDI being 18 nm and 0.228, respectively. More recently, by using oregano essential oil, Tween 80, ethanol, and deionized water, a resveratrol nanoemulsion with particle size at 137.5 nm and PDI at 0.22 was prepared [21]. By comparison, the mean particle size of the resveratrol nanoemulsion prepared in our study was smaller, which should be due to the difference in components used for its preparation. In addition, this resveratrol nanoemulsion showed a high stability as evident by the zeta potential being −49.7 mV based on a report by Shnoudeh et al. [22], stating that the zeta potential should be >30 mV or <−30 mV to maintain a high nanoemulsion stability. Also, the encapsulation efficiency was 95.5%, which was higher than that (93.4%) reported by Zhou et al. [23], who prepared a resveratrol nanoemulsion composed of soybean oil, lecithin, poloxamer 188, Labrasol, and deionized water.

### 3.3. Stability of Resveratrol Nanoemulsion

Only a slight change in particle size, PDI, and zeta potential was shown in the resveratrol nanoemulsion over a 90-day storage period at 4 °C, with the values ranging from 12.0 to 13.6 nm, 0.150 to 0.296, and −41.6 to −57.4 mV, respectively, revealing a high stability of this resveratrol nanoemulsion at low temperature. Although the zeta potential values varied from −41.6 to −57.4 mV during 90-day storage at 4 °C, it did not affect the stability of the resveratrol nanoemulsion, as a zeta potential value >30 mV or <−30 mV was used as an indicator of high stability as mentioned above [22]. However, during heating at 40–100 °C for 0.5–2 h, a slight change in particle size from 12.0 to 17.1 nm occurred (Table S1, see Supplementary Materials), while a pronounced decrease in zeta potential was observed following heating at 100 °C for >0.5 h. Thus, the heating temperature has to be controlled at 80 °C to maintain a high thermal stability of this resveratrol nanoemulsion.

### 3.4. Characteristics of GNPs and R-GNPs

**Figure 2:**
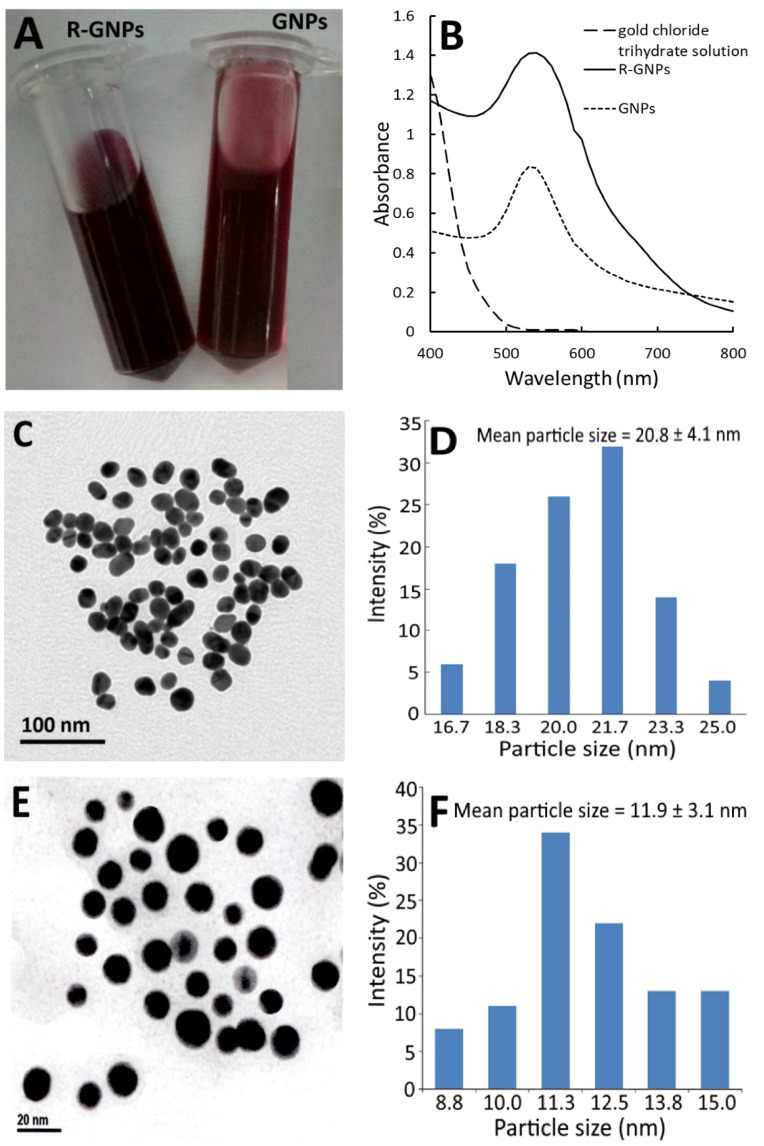
Appearance of gold nanoparticles (GNPs) and resveratrol-gold nanoparticles (R-GNPs) (**A**) and their absorption spectra showing the surface plasmon resonance peak, respectively, at 528 and 538 nm (**B**) as well as the transmission microscopic image obtained at 120 kV (**C**,**E**) along with particle size distribution (**D**,**F**) for GNPs (**C**,**D**) and R-GNPs (**E**,**F**).

Figure 2 shows the red appearance of both the GNPs and R-GNPs (Figure 2A) as well as the UV–VIS spectra of the gold chloride trihydrate solution, gold nanoparticle (GNPs), and resveratrol-gold nanoparticles (R-GNPs), with the maximum absorption wavelength of the surface plasmon resonance (SPR) peak of the GNPs and R-GNPs being 528 and 538 nm, respectively (Figure 2B). Similar findings were reported for resveratrol-gold nanoparticles by Tomoaia et al. [24], Park et al. [25], and Zhang et al. [26], showing the maximum absorbance of the SPR peaks to be 538, 540, and 539 nm, respectively. Also, a high stability of the GNPs and R-GNPs was shown as evident by a zeta potential of −32.7 mV and −66.7 mV, respectively. The TEM images of the GNPs and R-GNPs are shown in Figure 2C,E, respectively, with the mean particle size being 20.8 nm (Figure 2D) for the former and 11.9 nm (Figure 2F) for the latter, as well as a round shape for both. The particle size of the R-GNPs was smaller than that reported by Tomoaia et al. [24], Park et al. [25], and Zhang et al. [26], who reported the particle size of R-GNPs to be 50, 22.28, and 39 nm, respectively. This difference may be caused by the variation in preparation steps and conditions.

### 3.5. Tolerance of Solvent and Blank Nanoemulsion towards Cells

**Figure 3:**
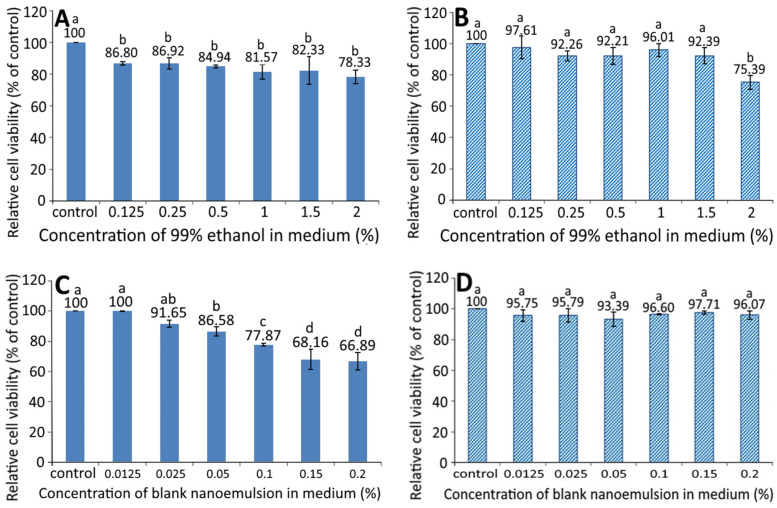
Effect of different concentrations of 99% ethanol and blank nanoemulsion (without resveratrol) on the growth of pancreatic normal cells MS1 (**A**,**C**) and pancreatic cancer cells BxPC-3 (**B**,**D**) after 48 h incubation as measured by MTT assay. Control represents that cells were incubated in RPMI medium only. Data are shown as mean ± standard deviation (*n* = 3) and data with different small letters (a–d) are significantly different at *p* < 0.05.

Figure 3 shows the effect of different concentration of 99% ethanol on the growth of pancreatic normal cells MS1 (**A**) and pancreatic cancer cells BxPC-3 (**B**). A dose-dependent decrease in the cell viability of MS1 was shown following treatment with 99% ethanol from 0.125 to 2%; however, there was no significant difference (*p* > 0.05) among the six doses. Similarly, a dose-dependent decline in the cell viability of BxPC-3 was observed following treatment with 99% ethanol from 0.125 to 1.5%, and there was a significant difference (*p* < 0.05) between 2% ethanol (99%) and the other five doses. By comparison at the same dose, 99% ethanol showed a higher inhibition effect against MS1 than BxPC-3 cells, implying that 99% ethanol at a dose from 0.125 to 1.5% possessed a slight toxicity towards MS1 cells, but not BxPC-3 cells.

The growth of MS1 and BxPC-3 cells as affected by different concentrations of blank nanoemulsions, is shown in Figure 3C,D, respectively. A dose-dependent decline in the cell viability of MS1 was shown following treatment with blank nanoemulsion (in medium) at a dose from 0.0125 to 0.2% and the decrease became significant (*p* < 0.05) after the level reached 0.05% and above as compared to the control. However, for BxPC-3 cells, the viability showed no significant difference (*p* > 0.05) between the control and the other six doses (0.0125–0.2%). Comparatively, the blank nanoemulsion showed a higher toxicity against MS1 cells than BxPC-3 cells at the same dose. Thus, both doses of 99% ethanol and the blank nanoemulsion were, respectively, controlled at 1.5% and 0.05% for MS1 cells as well as 1.5% and 0.2% for BxPC-3 cells for subsequent experiments.

### 3.6. Effects of Different Concentrations of the GNPs, R-GNPs, and Resveratrol Nanoemulsion on the Growth of MS1 and BxPC-3 Cells

**Figure 4:**
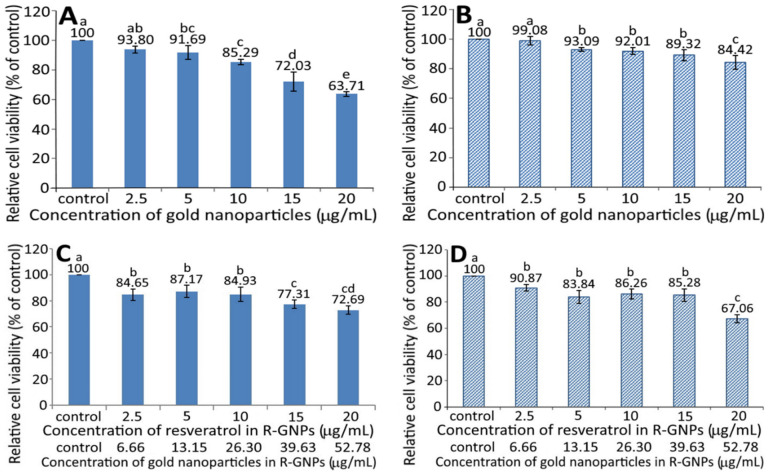
Effect of different concentrations of gold nanoparticles (GNPs) (**A**,**B**) and resveratrol-gold nanoparticles (R-GNPs) (**C**,**D**) on the growth of pancreatic normal cells MS1 (**A**,**C**) and pancreatic cancer cells BxPC-3 (**B**,**D**) after 48 h incubation as measured by MTT assay. Control represents that cells were incubated in RPMI medium only. Data are shown as mean ± standard deviation (*n* = 3) and data with different small letters (a–e) are significantly different at *p* < 0.05.

**Figure 5:**
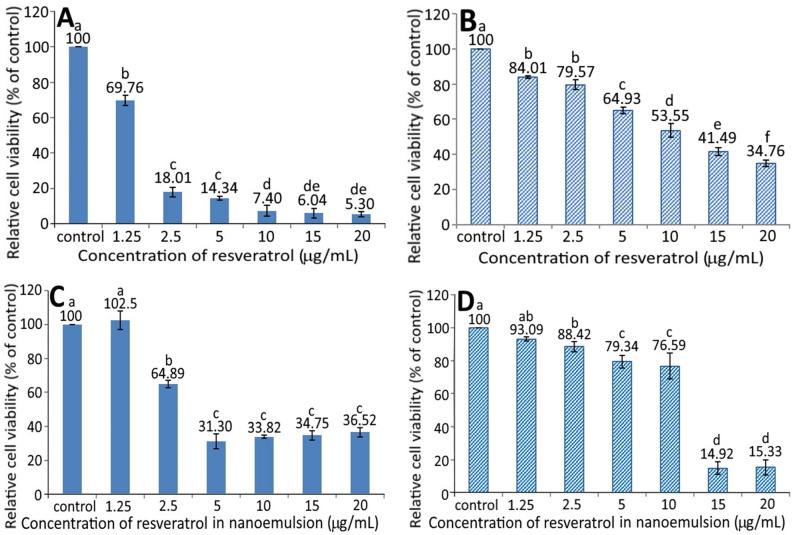
Effect of different concentrations of resveratrol standard (**A**,**B**) and resveratrol nanoemulsion (**C**,**D**) on the growth of pancreatic normal cells MS1 (**A**,**C**) and pancreatic cancer cells BxPC-3 (**B**,**D**) after 48 h incubation as measured by MTT assay. Control represents that cells were incubated in RPMI medium only. Data are shown as mean ± standard deviation (*n* = 3) and data with different small letters (a–f) are significantly different at *p* < 0.05.

Figure 4 shows the effect of different concentrations of GNPs (A and B) and R-GNPs (C and D) on the growth of pancreatic normal cells MS1 (A and C) and pancreatic cancer cells BxPC-3 (B and D). A dose-dependent decrease in viability was shown for both MS1 (Figure 4A) and BxPC-3 (Figure 4B) cells following treatment with the GNPs at a dose from 2.5 to 20 µg/mL. By comparison, at the same dose, the GNPs showed a higher toxicity against MS1 cells than BxPC-3 cells. Also, the difference between the control and GNPs at 2.5 µg/mL was not significantly different (*p* > 0.05) for either MS1 or BxPC-3 cells; however, this difference became significant (*p* < 0.05) after the level of GNPs reached 5% and above. Likewise, a dose-dependent decline in viability was shown for both MS1 and BxPC-3 cells following treatment with the R-GNPs at a dose from 2.5 to 20 µg/mL (resveratrol) and 6.66 to 52.78 µg/mL (gold). Also, the difference between the control and the other five doses was significantly different (*p* < 0.05) for both MS1 and BxPC-3 cells. By comparison at the same dose (2.5, 10, and 15 µg/mL of resveratrol and 6.66, 26.3, and 39.63 µg/mL of gold), the R-GNPs showed a higher toxicity against MS1 cells than BxPC-3 cells, but a reversed trend occurred for the doses at 5 and 20 µg/mL of resveratrol as well as at 13.15 and 52.78 µg/mL of gold. Compared to the GNPs at the same dose, the incorporation of resveratrol into the GNPs seemed to decrease the viability of both MS1 and BxPC-3 cells, indicating that resveratrol may possess a higher toxicity towards both MS1 and BxPC-3 cells than GNPs. This phenomenon was further confirmed in Figure 5A,B, showing a pronounced dose-dependent decline in the viability of both MS1 and BxPC-3 cells following the treatment of resveratrol standard at 1.25–20 µg/mL. Compared to BxPC-3 cells, resveratrol standard showed a much higher inhibition effect against MS1 cells at the same dose. However, this inhibition effect was drastically reduced for MS1 cells following treatment with the resveratrol nanoemulsion at the same dose (Figure 5C), revealing that the nanoemulsion composition, such as soybean oil and sucrose ester, may provide a protective effect towards MS1 cells. Also, resveratrol standard was more effective than the resveratrol nanoemulsion in inhibiting MS1 cells, as evident by an IC_50_ of 1.73 and 3.75 µg/mL, respectively. However, for BxPC-3 cells, the resveratrol nanoemulsion could elevate viability at a dose from 1.25–10 µg/mL as compared to resveratrol standard (Figure 5D), demonstrating again the protective effect of soybean oil and sucrose ester towards BxPC-3 cells. Collectively, resveratrol standard may exhibit a higher toxicity towards pancreatic normal cells MS1 than pancreatic cancer cells BxPC-3. Nevertheless, the resveratrol nanoemulsion was more effective than resveratrol standard in inhibiting BxPC-3 cells as evident by an IC_50_ of 11.02 and 13.75 µg/mL, respectively.

In published reports, most studies are focused on the effect of resveratrol standard on the inhibition of cancer cells. For instance, Cui et al. [27] studied the effect of resveratrol standard (5–200 µM) on the inhibition of pancreatic cancer cells AsPC-1, BxPC-3, and PANC-1 and the IC_50_ was shown to be 78.3 µM (17.87 µg/mL), 76.1 µM (17.37 µg/mL), and 123.3 ± 6.5 µM (28.14 µg/mL), respectively. For some other types of cancer cells, the IC_50_ was 24.74 µg/mL for hepatoma cells HepG2 following treatment with resveratrol standard (1–16 µg/mL). However, no toxicity was shown towards breast cancer cells MDA-MB-231 and MCF-7/adr in the presence of resveratrol standard at 50 µM (11.41 µg/mL) [28]. Interestingly, a higher resveratrol dose at 100 µM (22.82 µg/mL) decreased the viability of MCF-7/adr cells, while MDA-MB-231 cells remained unaffected [28]. In another study, Zhang et al. [26] studied the effect of resveratrol standard (1–16 µg/mL) on the growth of normal liver cells L02, and an inhibition percentage of 83% was shown after 24 h of incubation. However, the inhibition percentage decreased to <35% following treatment with resveratrol-nanogold, implying a lower toxicity towards L02 cells after the conjugation of nanogold with resveratrol standard. This outcome is similar to our finding that the resveratrol nanoemulsion was less toxic to pancreatic normal cells MS1 than resveratrol standard as evident by a higher IC_50_ of the former. Furthermore, compared to several previous studies shown above, the resveratrol nanoemulsion prepared in our study was more effective in inhibiting pancreatic cancer cells BxPC-3 than resveratrol standard as shown by a much lower IC_50_ of the former. Nevertheless, the cytotoxicity of resveratrol towards several other types of normal cells such as keratinocytes [29], smooth muscle cells [30], and endothelial cells [31] cannot be ignored.

As shown above, the resveratrol and resveratrol nanoemulsion were more effective in inhibiting both pancreatic normal cells MS1 and cancer cells BxPC-3 in comparison to the GNPs and R-GNPs (Figure 4 and Figure 5). This can be explained based on the possible activation of cell growth through localized extracellular interactions between gold nanoparticles and transmembrane signal receptors. Cancer is a complex and highly heterogeneous disease with various multidimensional factors being involved in cell growth via genomic changes. Among various factors, the dynamic interaction between cytoskeletal structures and a continuously changing extracellular matrix (ECM) plays a pivotal role in altering the genomic programming [32,33]. These interactions are caused by stress on integrin-like mechanosensors from multiple cellular forces and thereby activate cells’ membrane focal adhesion proteins and transmembrane signal receptors [34]. Most importantly, the mechanosensors regulate cancer cell growth by triggering many phosphorylation reactions through signal transaction between a cell’s extracellular active domain and intracellular F-actin filaments [35]. Consequently, it is possible that a treatment of cancer cells with nanoparticles can induce electrical polar interactions between nanoparticles in ECM and cells’ mechanosensors to facilitate cancer cell growth. In a study dealing with the effect of rare-earth fluoride nanoparticles (RFNPs) on three human cancer cells (lung cancer cell A549, colon cancer cell SW837, and breast cancer cell MCF7), Semashko et al. [35] reported that unlike large RFNPs (>20 nm), both tiny RFNPs (<10 nm) and small RFNPs (10–20 nm) could stimulate the growth of cancer cells by electrical dipole interactions between RFNPs in ECM and mechanosensors. Moreover, such electrical interactions were shown to be inversely proportional to square power of RFNPs’ size and depend on the electrical surface charge on both RFNPs and specific cell binding sites as well as their separating distance [35].

Thus, in our study, a higher cell viability of pancreatic cancer cells BxPC-3 for the GNPs and RGNPs compared to that for the resveratrol nanoemulsion (Figure 4 and Figure 5) may have been caused by electrical polar interactions between the gold nanoparticles (≤20 nm) and specific cellular binding sites promoting the activation of membrane receptors and protein expression. Also, the smaller the particle size, the higher the ability to promote cell growth. However, in our study R-GNPs with particle size at 11.9 nm was shown to inhibit BxPC-3 cancer cell growth to a higher extent than the relatively larger-sized GNPs (20.8 nm). A different size effect was shown for Herceptin-coated gold nanoparticles (H-GNPs) on breast cancer cells SK-BR-3, with 40 and 50 nm-sized H-GNPs showing a higher inhibition efficiency than H-GNPs at sizes <40 nm (2, 10, and 25 nm) [36]. Conversely, a similar trend was reported by Liu et al. [37], demonstrating that the small-sized citrate-capped GNPs (5 and 10 nm) could cause greater cytotoxicity towards lung cancer cells A549 and 95D than the large-sized ones (20 and 40 nm), implying that both nanoparticle size and cell-type are essential determinants that can selectively affect the cell proliferation, apoptosis, cell cycle, and cell invasion. Collectively, these findings reveal that nanoparticles can actively engage in mediating molecular processes for regulating cell functions, which can be dependent upon nanoparticle size, morphology, surface charge, coating material, concentration, and composition, as well as their strength of molecular bonding with cells and cell phenotypes [35,38,39].

### 3.7. Protein Expression Associated with Apoptosis

**Figure 6:**
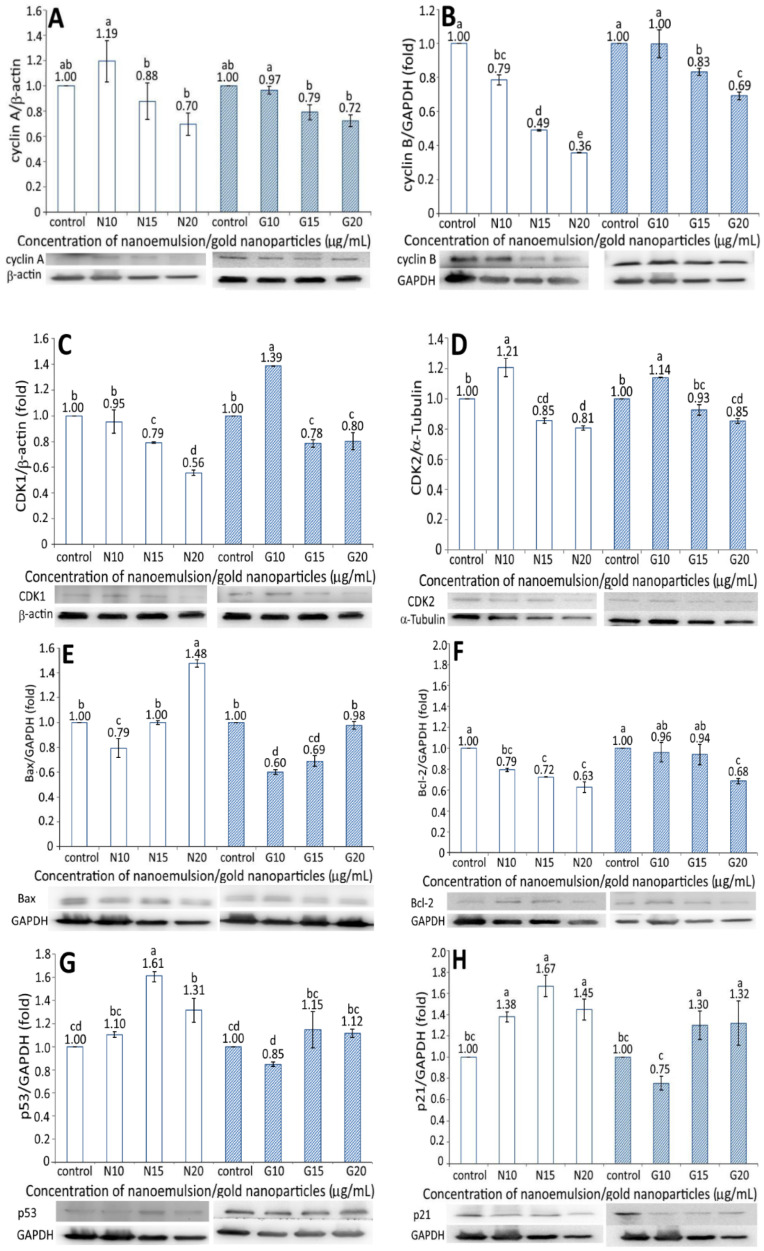

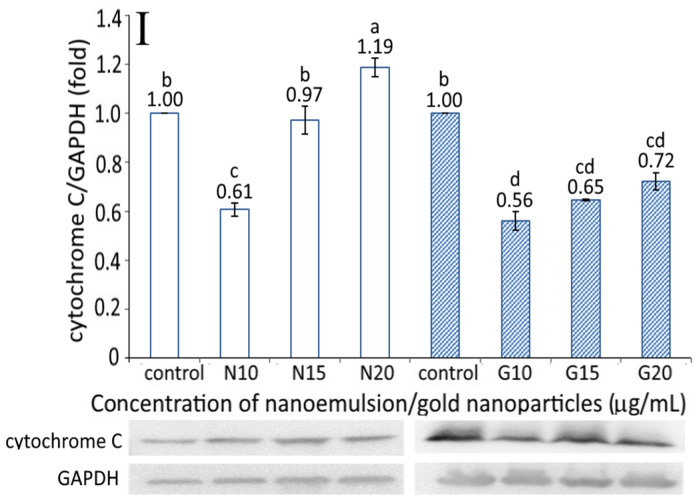
Effect of different concentrations (10, 15, and 20 μg/mL) of resveratrol nanoemulsion (N) and resveratrol-gold nanoparticles (G) on cyclin A (**A**), cyclin B (**B**), CDK1 (**C**), CDK2 (**D**), Bax (**E**), BcL-2 (**F**), p53 (**G**), p21 (**H**), and cytochrome C (**I**) expressions in pancreatic cancer cells BxPC-3. Control represents that cells were incubated in RPMI medium containing blank nanoemulsion without resveratrol (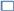) and gold nanoparticles without resveratrol (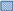). Data are shown as mean ± standard deviation (*n* = 3) and data with different small letters (a–e) are significantly different at *p* < 0.05.

The expression of proteins associated with the apoptosis of BxPC-3 cells, including cyclin A, cyclin B, CDK1, CDK2, Bax, BcL-2, p53, p21, and cytochrome C, is shown in Figure 6. A dose-dependent decrease in the cyclin A expression of BxPC-3 cells was shown for both the resveratrol nanoemulsion and R-GNPs treatments at a dose of 10–20 µg/mL (Figure 6A). Compared to the control, the resveratrol nanoemulsion (10 µg/mL) increased cyclin A expression by 1.19-fold but decreased it by 0.88- and 0.7-fold at a dose of 15 and 20 µg/mL, respectively, while the difference between 10 µg/mL and the other two doses was significantly different (*p* < 0.05). Similarly, compared to the control, the R-GNPs treatment decreased the cyclin A expression by 0.97-, 0.79-, and 0.72-fold at a dose of 10, 15, and 20 µg/mL, respectively, while the difference between 10 µg/mL and the other two doses was significantly different (*p* < 0.05). Comparatively, both the resveratrol nanoemulsion and R-GNPs showed a similar inhibition effect on the cyclic A expression at the same dose. Likewise, a dose-dependent decline in the cyclin B expression was shown for both the resveratrol nanoemulsion and R-GNPs treatment at a dose from 10 to 20 µg/mL (Figure 6B). However, a different trend occurred for both treatments when compared at the same dose, as evident by a more pronounced inhibition effect on the cyclin B expression following treatment with the resveratrol nanoemulsion. Moreover, compared to cyclin A, the resveratrol nanoemulsion showed a much higher inhibition effect on the cyclin B expression at the same dose, while the R-GNPs showed a similar inhibition effect. Accordingly, cyclin A is mainly responsible for DNA replication (S phase), while cyclin B is for the preparation of materials for mitosis (G2 phase) to proceed. Thus, a dose-dependent decline in the cyclin A expression may indicate that the DNA replication was gradually phased out following treatment with the resveratrol nanoemulsion. Similarly, a dose-dependent decrease in the cyclin B expression may imply that mitosis was retarded following the same treatment. In other words, pancreatic cancer cells BxPC-3 were retarded to a greater extent at G2 phase than at S phase.

Figure 6C,D shows the CDK1 and CDK2 expressions in BxPC-3 cells as affected by both the resveratrol nanoemulsion and R-GNPs, respectively. Like cyclin A and cyclin B, a dose-dependent decrease in the CDK1 and CDK2 expressions of BxPC-3 cells was shown for both the resveratrol and R-GNPs treatments. Also, by comparison at the same dose, the resveratrol nanoemulsion showed a higher inhibition effect on the CDK1 expression than the R-GNPs. However, for the CDK2 expression, both the resveratrol nanoemulsion and R-GNPs treatments showed a similar inhibition effect as no significant difference (*p* > 0.05) was shown when compared at the same dose. Furthermore, compared to CDK2, the resveratrol nanoemulsion showed a much higher inhibition effect on the CDK1 expression at the same dose, while the R-GNPs only showed a similar inhibition effect.

For the Bax and BcL-2 expressions, a dose-dependent increase was shown for the former (Figure 6E) and a decrease for the latter (Figure 6F). By comparison, the Bax expression was raised to a much higher level for the resveratrol nanoemulsion than for the R-GNPs at the same dose, implying that the former was more efficient than the latter in elevating the Bax expression. However, a different trend was observed for the BcL-2 expression as a dose-dependent decline occurred for both the resveratrol nanoemulsion and R-GNPs treatments. Unlike the Bax expression, the resveratrol nanoemulsion showed a higher inhibition effect than the R-GNPs in inhibiting the BcL-2 expression at the same dose. This outcome clearly revealed that the resveratrol nanoemulsion was more effective than the R-GNPs in inhibiting the growth of BxPC-3 cells through the elevation of Bax expression, a pro-apoptotic protein, and the inhibition of BcL-2 expression, an anti-apoptotic protein. A similar outcome was reported by Cui, et al. [27], who studied the effect of resveratrol standard (0–200 µM) on pancreatic cancer cells PAN-1, BxPC-3, and AsPC-3; a dose-dependent rise in the Bax expression and decline in the BcL-2 expression were shown.

Figure 6G and Figure 6H show the expressions of p53 and p21 in BxPC-3 cells, respectively, as affected by both the resveratrol nanoemulsion and R-GNPs. Compared to the control, only two doses of the resveratrol nanoemulsion at 15 and 20 µg/mL could enhance the p53 expression by 1.61- and 1.31-fold, respectively. The same trend was shown for the R-GNPs treatment at the same dose, with the p53 expression being raised by 1.15- and 1.12-fold as compared with the control. Comparatively, the resveratrol nanoemulsion could elevate the p53 expression to a higher level than the R-GNPs at the same dose. Similar findings were observed for the p21 expression, with the level being raised by 1.38-, 1.67-, and 1.45-fold following treatment with the resveratrol nanoemulsion at 10, 15, and 20 µg/mL, respectively, as compared to the control. For the R-GNPs treatment, the p21 expression was raised by 1.30- and 1.32-fold at a dose of 15 and 20 µg/mL, respectively. By comparison, the resveratrol nanoemulsion could elevate both the p53 and p21 expressions to a higher level than the R-GNPs at the same dose. Furthermore, following treatment with the resveratrol nanoemulsion or R-GNPs, the p21 expression was raised to a higher level than the p53 expression at 15 and 20 µg/mL. In several previous studies, Zhou et al. [40] studied the effect of resveratrol standard (0–200 µM) on the inhibition of pancreatic cancer cells capan-2 and colo-357 and reported a rise of both the p53 and p21 expressions, accompanied by an increase of caspase-3 activity to promote cell apoptosis. 

The cytochrome C expression as affected by the resveratrol nanoemulsion and R-GNPs is shown in Figure 6I. A dose-dependent increase in the cytochrome C expression was shown for both the resveratrol and R-GNPs treatments. By comparison at the same dose, the resveratrol nanoemulsion was more effective in elevating the cytochrome C expression than the R-GNPs. This outcome also implies that resveratrol nanoemulsions could facilitate cytochrome C release from mitochondria to form a complex with pro-caspase-9 for caspase-9 activation to initiate cell apoptosis [14].

### 3.8. Activities of Caspase-3, Caspase-8, and Caspase-9

**Figure 7:**
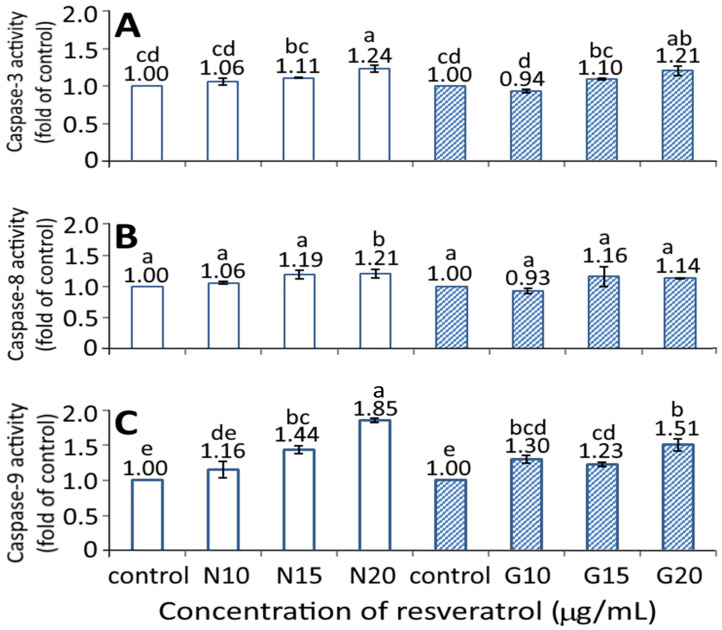
Effect of different concentrations (10, 15, and 20 μg/mL) of resveratrol nanoemulsion and resveratrol-gold nanoparticles on caspase-3 (**A**), caspase-8 (**B**), and caspase-9 (**C**) activities of pancreatic cancer cells BxPC-3. Control represents that cells were incubated in RPMI medium containing blank nanoemulsion without resveratrol (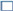) and gold nanoparticles without resveratrol (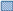). N and G represent resveratrol nanoemulsion and resveratrol-gold nanoparticles, respectively. Data are shown as mean ± standard deviation (*n* = 3) and data with different small letters (a–e) are significantly different at *p* < 0.05.

Figure 7 shows the effect of the resveratrol nanoemulsion and R-GNPs on activities of caspase-3, caspase-8, and caspase-9. A dose-dependent rise in activities of caspase-3, caspase-8, and caspase-9 was shown for both the resveratrol nanoemulsion and R-GNPs treatments. However, by comparison at the same dose (15 and 20 µg/mL), the resveratrol nanoemulsion showed a significantly higher (*p* < 0.05) caspase-9 activity than the R-GNPs, but no significant difference (*p* > 0.05) was observed for either the caspase-3 or caspase-8 activities. Accordingly, cell apoptosis can be regulated through death receptor, mitochondria, or endoplasmic reticulum pathways, in which the caspase protein plays a vital role in cell apoptosis. More specifically, following a decrease in BcL-2 expression, cytochrome C can be released from mitochondria to form a complex with pro-caspase-9 for caspase-9 activation for apoptosis initiation through the mitochondria pathway. In addition, caspase-8 can be activated through the death receptor pathway for the initiation of apoptosis, while caspase 3 can be activated through both mitochondria and death receptor pathways for the execution of apoptosis. As a high dose (20 µg/mL) of both the resveratrol nanoemulsion and R-GNPs showed a significantly higher (*p* < 0.05) caspase-3 activity than that of the control, this finding suggests that both death receptor and mitochondria pathways may be responsible for the apoptosis of BxPC-3 cells. In several similar studies, Mouria et al. [41] also reported that both cytochrome C and caspase-3 activities were elevated following the treatment of pancreatic cancer cells Mia-PACA-2 with resveratrol standard (25 µM). Similarly, the caspase-3 and PARP activities were raised following the treatment of pancreatic cancer cells PANC-1 and BxPC-3 with resveratrol standard (50 µM) [42]. A dose of resveratrol standard (50 µM) could raise activities of caspase-3, caspase-8, and caspase-9 in both pancreatic cancer cells PANC-1 and BxPC-3, while a dose of 100 µM could also raise all the caspase activities in pancreatic cancer cells AsPC-3 [27]. Additionally, both Bax and caspase-3 expressions were enhanced while BcL-2 expression decreased following the treatment of pancreatic cancer cells MiaPaCa-2 with resveratrol standard at 100 μM [43].

## 4. Conclusions

Collectively, in our study both the resveratrol nanoemulsion prepared from grape skin and the R-GNPs from gold and resveratrol standard could inhibit the growth of pancreatic cancer cells BxPC-3 through the inhibition of cyclin A, cyclin B, CDK1, and CDK2 expressions and the elevation of both p53 and p21 expressions. Meanwhile, the Bax expression was enhanced and BcL-2 expression inhibited to reduce mitochondria membrane potential for cytochrome C release from mitochondria for the activation of both caspase-8 and caspase-9 for initiation of the apoptosis, followed by the activation of caspase-3 for the execution of apoptosis of BxPC-3 cells. Further research is necessary to study the inhibition of pancreatic tumors in vivo by resveratrol nanoemulsions and R-GNPs.

## Data Availability

Not applicable.

## Supplementary Materials

The following are available online at https://www.mdpi.com/article/10.3390/pharmaceutics13111871/s1, Table S1: Changes in particle size and zeta potential of resveratrol nanoemulsion during heating at 40–100 °C for varied time length.
