# Supplementary material for: Comparative Study on Inhibition of Pancreatic Cancer Cells by Resveratrol Gold Nanoparticles and a Resveratrol Nanoemulsion Prepared from Grape Skin
Source: Pharmaceutics. 2021 Nov 5;13(11):1871. doi: 10.3390/pharmaceutics13111871 (PMC8622665; doi:10.3390/pharmaceutics13111871)
Supplement: Supplementary file 1 [file pharmaceutics-13-01871-s001.zip › pharmaceutics-1398683-supplementary.pdf]

pharmaceutics

MDPI

Supplementary Materials: Comparative Study on Inhibition of Pancreatic Cancer Cells by Resveratrol Gold Nanoparticles and a Resveratrol Nanoemulsion Prepared from Grape Skin

Baskaran Stephen Inbaraj, Leng-Huei Hua and Bing-Huei Chen

Table S1. Changes in particle size and zeta potential of resveratrol nanoemulsion during heating at 40–100 °C for varied time length.

Temperature (°C)	Particle size (nm)					Zeta potential (mV)				
	Heating time					Heating time				
	0 h	0.5 h	1 h	1.5 h	2 h	0 h	0.5 h	1 h	1.5 h	2 h
40	14.1 ± 3.4	13.9 ± 1.8	14.4 ± 2.1	13.5 ± 5.3	14.9 ± 2.9	−69.0 ± 2.9	−62.1 ± 1.5	−60.8 ± 0.9	−54.6 ± 1.8	−60.5 ± 1.9
60	14.1 ± 3.4	13.0 ± 1.3	12.0 ± 2.5	13.2 ± 3.1	15.6 ± 1.5	−69.0 ± 2.9	−59.3 ± 1.6	−58.3 ± 0.2	−56.7 ± 1.3	−51.8 ± 0.3
80	14.1 ± 3.4	13.3 ± 4.9	13.8 ± 3.1	12.7 ± 3.7	15.8 ± 0.3	−69.0 ± 2.9	−51.3 ± 1.1	−57.5 ± 2.7	−50.0 ± 1.9	−51.2 ± 2.9
100	14.1 ± 3.4	15.3 ± 2.8	17.9 ± 0.4	17.0 ± 4.8	17.1 ± 3.4	−69.0 ± 2.9	−29.8 ± 3.2	−26.7 ± 4.1	−23.8 ± 2.6	−21.6 ± 0.2
